# Predictors of health-related quality of life for children with neurodevelopmental conditions

**DOI:** 10.1038/s41598-024-56821-9

**Published:** 2024-03-16

**Authors:** Maryam Mahjoob, Robyn Cardy, Melanie Penner, Evdokia Anagnostou, Brendan F. Andrade, Jennifer Crosbie, Elizabeth Kelley, Muhammad Ayub, Muhammad Ayub, Jessica Brian, Alana Iaboni, Russell Schachar, Stelios Georgiades, Rob Nicolson, Jessica Jones, Azadeh Kushki

**Affiliations:** 1 Institute of Biomedical Engineering, University of Toronto, Toronto, Canada; 2 Bloorview Research Institute, Holland Bloorview Kids Rehabilitation Hospital, 150 Kilgour Road, Toronto, ON M4G 1R8 Canada; 3 Department of Psychiatry, Margaret and Wallace McCain Centre for Child Youth and Family Mental Health, Centre for Addiction and Mental Health, University of Toronto, Toronto, Canada; 4 Department of Psychiatry, The Hospital for Sick Children (SickKids), Toronto, Canada; 5 Department of Psychology, Queen’s University, Kingston, Canada; 6 Department of Psychiatry, Queen’s University, Kingston, Canada; 7 Psychiatry & Behavioural Neurosciences, McMaster University, Hamilton, Canada; 8 Department of Psychiatry, Western University, London, Canada

**Keywords:** Quality of life, Neurodevelopmental disorders, Structural equation modelling, Paediatric research, Quality of life

## Abstract

Neurodevelopmental conditions can be associated with decreased health-related quality of life; however, the predictors of these outcomes remain largely unknown. We characterized the predictors of health-related quality of life (HRQoL) in a sample of neurodiverse children and youth. We used a cross-sectional subsample from the Province of Ontario Neurodevelopmental Disorders Network (POND) consisting of those children and young people in the POND dataset with complete study data (total n = 615; 31% female; age: 11.28 years ± 2.84 years). Using a structural equation model, we investigated the effects of demographics (age, sex, socioeconomic status), core features (Social Communication Questionnaire, Toronto Obsessive Compulsive Scale, Strengths and Weaknesses of attention deficit/hyperactivity disorder (ADHD)-symptoms and Normal Behavior), co-occurring symptoms (Child Behaviour Checklist), and adaptive functioning (Adaptive Behaviour Assessment System) on HRQoL (KINDL). A total of 615 participants had complete data for this study (autism = 135, ADHD = 273, subthreshold ADHD = 7, obsessive–compulsive disorder (OCD) = 38, sub-threshold OCD = 1, neurotypical = 161). Of these participants, 190 (31%) identified as female, and 425 (69%) identified as male. The mean age was 11.28 years ± 2.84 years. Health-related quality of life was negatively associated with co-occurring symptoms (B = − 0.6, SE = 0.20, CI (− 0.95, − 0.19), p = 0.004)) and age (B = − 0.1, SE = 0.04, CI (− 0.19, − 0.01), p = 0.037). Fewer co-occurring symptoms were associated with higher socioeconomic status (B = − 0.5, SE = − 0.05, CI (− 0.58, − 0.37), p < 0.001). This study used a cross-sectional design. Given that one’s experiences, needs, supports, and environment and thus HrQoL may change significantly over the lifespan and a longitudinal analysis of predictors is needed to capture these changes. Future studies with more diverse participant groups are needed. These results demonstrate the importance of behavioural and sociodemographic characteristics on health-related quality of life across neurodevelopmental conditions.

## Introduction

Autism spectrum disorder (ASD; autism), attention deficit/hyperactivity disorder (ADHD), and obsessive–compulsive disorder (OCD) are diagnostic labels referring to groups of neurodevelopmental conditions that manifest early in life and can be associated with differences in personal, social, occupational, and academic abilities^1,2^. These conditions are highly heterogeneous and have been associated with significant overlap in etiology, biology, and phenotype^3^. For example, a recent meta-analysis estimated the prevalence of ADHD and OCD in autism as 28% and 9%, respectively^4^. Furthermore, several neuroimaging studies have reported shared patterns of brain structure^3,5^ and function^6,7^ among these conditions. Constellation of the core and co-occurring autism, ADHD, and OCD features, combined with environmental and societal factors can significantly impact an individual’s function and overall quality of life (QoL)^8–10^.

QoL is a subjective construct defined as one’s satisfaction with life in relation to culture, value systems, goals, expectations, standards, and concerns^11^. Diagnoses of autism, ADHD, and OCD have been associated with decreases in health-related quality of life^12^ (Health-related QoL; HrQoL) for children with these conditions^13^. The significant heterogeneity within neurodevelopmental conditions, including variability in the strength and presentation of core and co-occurring symptoms^3^, translates to large variability in HrQoL outcomes^14^. Some of the factors that may contribute to this variability include individual differences in profiles of strengths and needs, and social, demographic, economic, and environmental factors; however, the contribution of these factors to HrQoL remains relatively unknown for children with neurodevelopmental conditions. Our recent systematic review revealed significant gaps in our understanding of predictors of HrQoL in neurodevelopmental conditions^15^. At the same time, the review found that many predictors of HrQoL transcend diagnosis boundaries (e.g., adaptive functioning, mental health), motivating a transdiagnostic approach.

In samples of children with diagnoses of autism, ADHD, and OCD, in particular, a handful of studies suggest that the extent of core-domain symptoms (social communication and repetitive behaviours in autism^9^; hyperactivity in ADHD^8^; obsessions and compulsions in OCD^16^) as well as adaptive functioning^9^ may decrease HrQoL. In addition to these domains, autism, ADHD, and OCD are also associated with increased risk of mental health symptoms including internalizing (inwardly focused behaviours including anxiety and depression) and externalizing behaviours (outwardly focused behaviours including impulsivity, rule breaking, and aggression)^13,17,18^, associated with decreased psychological well being^19,20^ and can negatively impact HrQoL^9,16,21^. However, studies examining the effect of these challenges on HrQoL in autism, ADHD, and OCD are limited, have small sample sizes, and only consider a subset of variables in isolation^10,15,22,23^.

Demographic variables and other social determinants of health can also impact HrQoL. Sex and gender may influence HrQoL through inequitable access to health services, social demands, and higher rates of mental health challenges in females^24^. Age is suggested to influence HrQoL through changes in age-related developmental^25^ and social experiences^26^. In samples of children with autism, ADHD, and OCD, studies examining the effects of age and sex and gender on HrQoL have reported mixed findings when comparing diagnosis groups^26–28^.

In addition to age and sex and gender, other social factors, such as race/ethnicity and socioeconomic status, can impact HrQoL directly through access to resources to improve well-being^29^ and experiences of oppression and discrimination^30^. These factors can also indirectly impact HrQoL through their influence on symptoms (e.g., access to supports). However, the effects of these determinants on overall HrQoL has limited investigation in children with autism^31^ or ADHD^22^ and no investigation in children with OCD.

Another critical limitation of the existing literature is the focus on diagnostic categories in isolation. Autism, ADHD, and OCD co-occur at high rates with significant overlap in symptoms^32–35^. There is emerging evidence to question the validity of these diagnostic labels as a result of the heterogeneity and suggestions for transdiagnostic approaches across autism, ADHD, and OCD based on clinical and neuroanatomical attributes^3^. From a dimensional perspective^36^, many symptom domains are also shared among these diagnoses. To this end, the goal of this study was to study the correlates of HrQoL for children with autism, ADHD, and OCD with a diagnosis-agnostic lens.

Based on the previous literature, we hypothesize that symptoms, co-occurring symptoms, and demographic variables are associated with HrQoL in neurodiverse groups.

## Methods

### Participants

This study conducted a secondary analysis of a subset of the data from the Province of Ontario Neurodevelopmental Disorders (POND) Network, consisting of children and youth with complete study data (Additional File 5). The data were exported on July 23, 2021, and contained a total of 1472 participants aged 7 to 17 years old (autism = 541, ADHD = 445, sub-threshold ADHD = 154, OCD = 119, sub-threshold OCD = 6, typically developing, TD = 207). This age range was chosen based on the availability of the HrQoL measure. Sub-threshold participants were included in part of the transdiagnostic approach of our analysis, as symptoms which do not meet clinical thresholds for a diagnosis may still influence quality of life. Sub-threshold participants were seen at the Hospital for Sick Children and received their designation through clinical judgement. Diagnoses were supported by gold-standard assessment: The Autism Diagnostic Observation Schedule (ADOS)^37^ and the Autism Diagnostic Interview -Revised (ADI-R) for autism^38^, Parent Interview for Child Symptoms (PICS) for ADHD^39^, and the Children’s Yale-Brown Obsessive Compulsive Scale for OCD^40^. The TD participants part of POND did not have a neurodevelopmental, psychiatric, or neurological condition, were not born prematurely and had no first-degree family history of a neurodevelopmental condition. Informed consent was provided by participants who were determined to have capacity to consent. Otherwise, consent was obtained from guardians and assent was provided by the participants. Research ethics approval was provided at each participating POND institution (Holland Bloorview Kids Rehabilitation Hospital, Hospital for Sick Children, McMaster University, McMaster Children’s Hospital, St Joseph’s Hospital, Western University, Children’s Hospital at London Health Sciences Centre, Queen’s University). All methods were performed in accordance with the relevant guidelines and regulations.

### Theoretical framework

**Figure 1:**
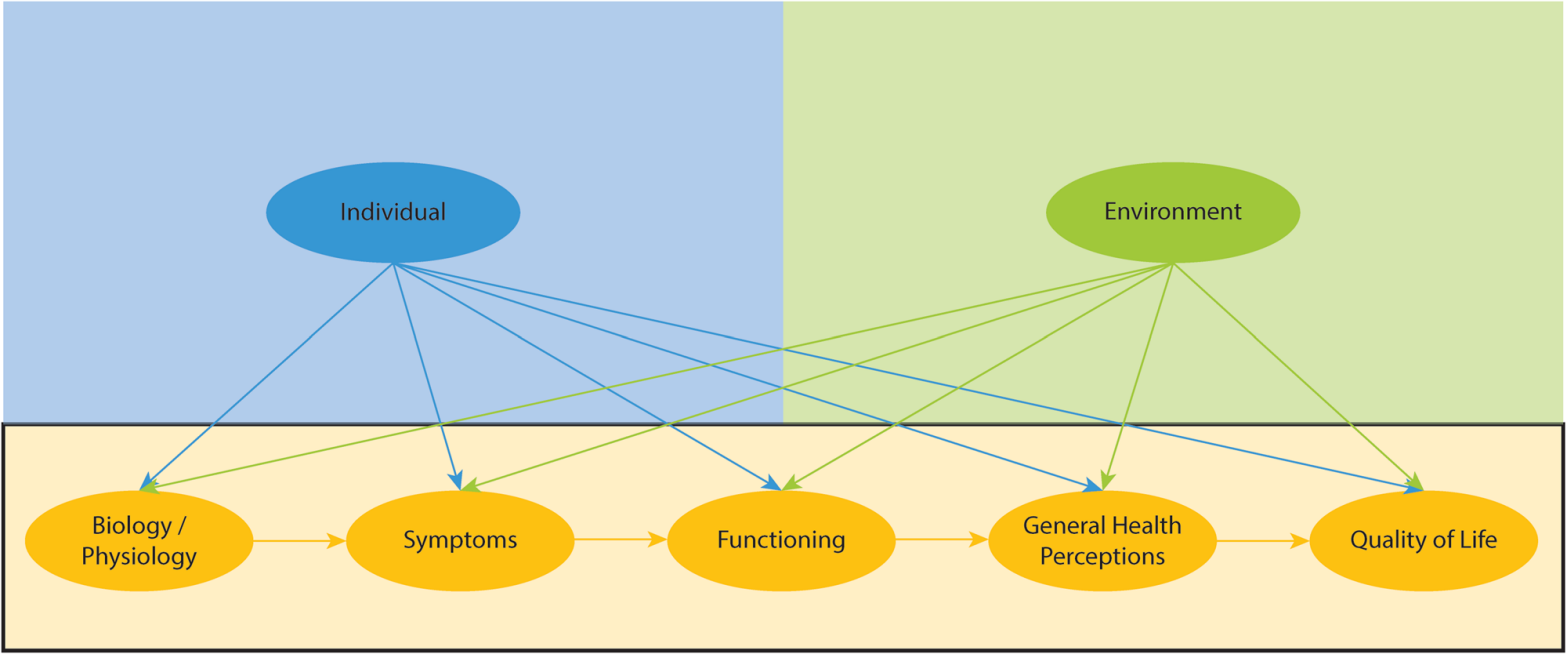
Revised Wilson and Cleary Model adapted from Ferrans et al.

Various conceptual models have been developed to theorize determinants of HrQoL. In this study, we built upon the revised version of the 1995 Wilson and Cleary Model made by Ferrans et al. in 2005^41^ (Fig. 1), to model the interrelationships among dimensions impacting HRQoL. According to a systematic review of 100 articles evaluating HrQoL, the revised version of the 1995 Wilson and Cleary Model was one of the most frequently and most comprehensive used models in HrQoL research^42^. The Wilson and Cleary model of HRQoL predictors includes seven domains. These include biological/physiological factors (variables that quantify the function of cells, organs, or body systems), symptoms (quantifies perception of a person’s physical, emotional, or cognitive state, measured on a global or condition/domain-specific measure, functioning (the ability to perform tasks across multiple domains), general health perceptions (subjective rating integrating previous health concepts), overall quality of life (subjective rating of wellbeing), and characteristics of the individual (demographic, development, psychological, and biological influences of health outcomes) and environment (social and physical influencers of health outcomes).

### Measures

**Figure 2:**
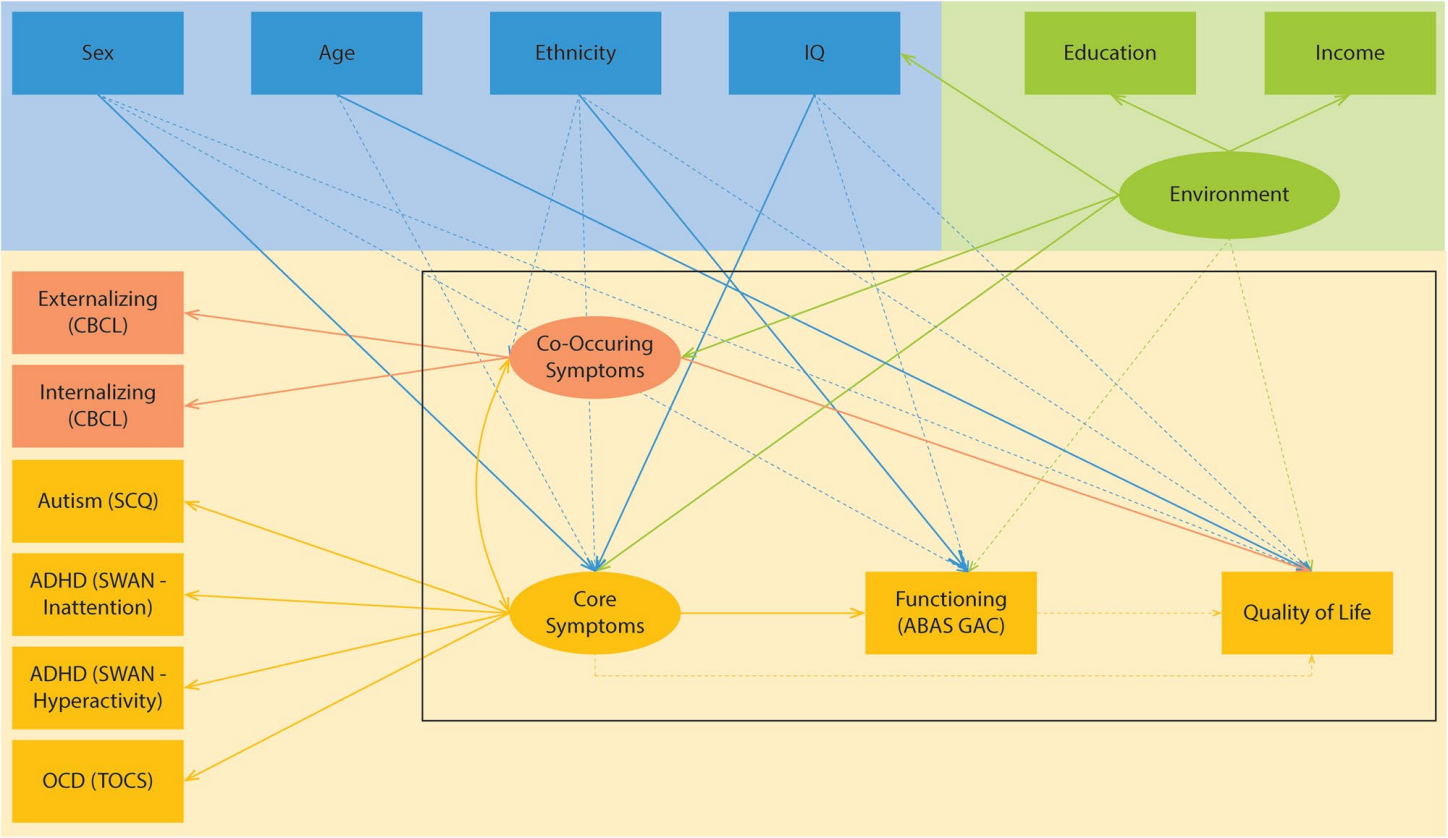
Adaptation of the Revised Wilson and Cleary Model.

We operationalized the Revised Wilson and Cleary model for use in our analyses as shown in Fig. 2. HrQoL was quantified using the KINDL^43^. The KINDL is a self- or parent-reported measure of HrQoL in clinical or non-clinical pediatric populations^43^. Our study used the self-reported KINDL, which included the Kid-KINDL (ages 7 to 13) and the Kiddo-KINDL (ages 14 to 17)^43^. The Kid- and Kiddo-KINDL contain 6 domains of physical well-being, emotional well-being, self-esteem, family, friends, and everyday functioning^43^. Each question is scored on a 5-point Likert scale (never, seldom, sometimes, often, all the time), with high scores indicating worse HrQoL. In a sample of clinical and non-clinical populations, the KINDL has demonstrated a high degree of internal consistency (Cronbach’s = 0.84) and a moderate convergent validity (r = 0.40–0.72) to similar instruments (e.g. Child Health Questionnaire and the SF-36).

For symptoms, core-domain features of autism, ADHD, and OCD as well as co-occurring domains of internalizing and externalizing symptoms were used. Autism traits were quantified using the Social Communication Questionnaire Lifetime Version (SCQ)^44^. ADHD symptoms were measured using the Strengths and Weaknesses of ADHD-symptoms and Normal Behavior (SWAN) rating scale^45^ for ADHD traits. OCD symptoms were characterized using the Toronto Obsessive Compulsive Scale (TOCS)^46^. Internalizing and externalizing symptoms were quantified using the subscales of the Child Behaviour Checklist (CBCL)^47^.

Functional abilities were assessed by the Adaptive Behaviour Assessment System (ABAS) parent-report questionnaire for ages 5 to 21^48^. The General Adaptive Composite (GAC) score, a standardized measure of conceptual, social, and practical scores, was used.

Individual factors included age, IQ, sex, and ethnicity. Full scale IQ was quantified using the Weschler Abbreviated Scale of Intelligence (first or second edition)^49^, the Weschler Intelligence Scale for Children (Fourth or Fifth Edition)^50^, the Stanford-Binet Intelligence Scale^51^, or the Weschler Preschool and Primary Scale of Intelligence (Fourth Edition)^52^. Ethnicity was modeled as a binary variable, white (European descent) versus non-white (Latin, Black, East Asian, Southeast Asia, Indigenous, Middle Eastern, and South Asian) due to our sample size and composition.

Environmental characteristics consisted of socioeconomic status (SES), which integrated information on the caregiver’s educational status and the household income. We hypothesized SES would be associated with all primary domains in the model, co-occurring symptoms, and IQ. Due to the well-established link between the influence of SES on mental health^53^ and on IQ^54^, a regression path between SES and these individual indicators was included. The domains of general health perceptions and biology/physiology were not attainable from the POND database, and thus were excluded from the model. Table 1 summarizes each variable in its appropriate domain, based on the revised Wilson and Cleary Model.

**Table 1:** Study variables categorized in domains of revised Wilson and Cleary model.

Individual	Environment	Symptoms	Functioning	HRQoL
Sex Age Ethnicity Full Scale IQ	Household Income Primary Caregiver Education	SCQ SWAN TOCS CBCL Internalizing CBCL Externalizing	ABAS General Composite Score	Kiddo/Kid KINDL

### Statistical analysis

To explore pairwise associations between variables, Pearson correlations were used. Alpha was set at 0.05, and P-values were corrected using the Holm-Bonferroni correction^55^.

To understand the correlates of HrQoL, we employed Structural equation modelling (SEM)^56^. SEM is a multivariate analysis technique that provides a flexible framework for analyzing complex associations among multiple measured and latent variables. Latent variables are unobserved variables whose values are inferred mathematically from the model. SEM can be used to test the validity of theoretical models using empirical data^57^. In this study, we used the revised Wilson and Cleary model to construct the SEM model (Fig. 2). In this model, environment and symptoms were constructed as latent variables. Participant-reported education and income were considered as indicators of environment as noted by direct paths among these variables. The symptom domain of the Wilson and Cleary model was conceptualized as core symptoms relevant to autism (SCQ), ADHD (SWAN), and OCD (TOCS) as well as co-occurring mental health symptoms (CBCL internalizing and externalizing symptoms). Five variables were included as individual factors, namely sex, age, ethnicity, and IQ. Finally, the functioning domain of the Wilson and Cleary model was quantified by the adaptive functioning measure in POND (ABAS II). In total, 14 measured variables and three latent variables were included in the model. The paths in the SEM model were informed by the Wilson and Cleary model as well as prior literature, including a recent systematic review^15^. In particular, core and co-occurring symptoms were linked to model the potential association among these domains^58,59^. Individual factors were connected to symptoms to model sex- and age-related differences in symptoms^60–63^ as well as diagnosis effects on prevalence of co-occurring intellectual disability. Individual factors were also connected to the functioning domain to reflect associations based on theoretical considerations in the Ferrans model^41^ as well as literature findings characterizing these associations^64–66^. Finally, the environment variable was connected to symptoms, functioning, and quality of life to reflect the impact of social determinants of health on these domains.

SEM analysis was completed using the lavaan package in R studio 4.1.2^67^. The measures (CBCL internalizing, CBCL externalizing, KINDL total score, the ABAS general adaptive composite, and IQ) were normalized prior to application of the SEM. Standardized coefficients were used to determine the relative contribution of each measure to the model. Acceptable model fit was determined based on the following: normed Chi-square value (< 5 for good model fit^68^); Comparative Fit Index (CFI) and Tucker Lewis-Index (TLI) (> = 0.95 indicating excellent model fit, >  = 0.9 indicating acceptable model fit^69,70^); Root Mean Square Error of Approximation (RMSEA) and Standard Root Mean Residual (SRMR) (< = 0.08 indicating good model fit, with values < 0.05 indicating excellent model fit^71^). The model was fitted with the diagonally weighted least square estimator (WLSMV) estimator^72^ due to the categorical data in the model. Based on commonly used estimates of 5–10 samples per parameter or 10 samples per variable, a sample size of 615 provides sufficient power for our analysis^73^.

### Ethics approal and conent to paticipate

The POND study was approved by each participating institution’s research ethics boards; written informed consent or assent was obtained from the primary caregiver or participant where appropriate.

## Results

### Participant demographics

A total of 615 children and youth had complete data (from the 1472 original participants, Additional File 1) and were included in the SEM models (Table 2). The Kid-KINDL was completed by 460 participants and the Kiddo-KINDL was completed by 155 participants (Additional File 2). Two participants reported a non-binary gender or not specified and were removed from the analyses given the small sample size.

**Table 2:** Participant characteristics.

	ADHD	Autism	OCD	SubADHD	SubOCD	TD	Total	Adjusted P-Value (group)^j^
	*N* = *135*	*N* = *273*	*N* = *38*	*N* = *7*	*N* = *1*	*N* = *161*	*N* = *615*	
Sex								< 0.001
Female (n (%))	41 (30%)	61 (22%)	17 (45%)	2 (29%)	1 (100%)	68 (42%)	190 (31%)	
Male (n (%))	94 (69%)	212 (78%)	21 (55%)	5 (71%)	0	93 (58%)	425 (69%)	
Age in years (mean ± standard deviation)	10.38 ± 2.54	11.63 ± 2.88	12.87 ± 2.72	9.86 ± 3.48	13.00	11.12 ± 2.78	11.28 ± 2.84	< 0.001
Income								< 0.001
High ($150,000) (n (%))	28 (20%)	59 (22%)	12 (32%)	3 (43%)	1 (100%)	65 (40%)	168 (27%)	
Middle ($50,000–$150,000)	62 (46%)	134 (49%)	18 (47%)	3 (43%)	0 (0%)	74 (46%)	291 (47%)	
Low ($0–$49,999)	45 (33%)	80 (29%)	8 (21%)	1 (14%)	0 (0%)	22 (14%)	156 (26%)	
Education								< 0.001
Graduate/professional (n (%))	19 (14%)	48 (18%)	9 (24%)	4 (57%)	1 (100%)	55 (34%)	136 (22%)	
Associate (n (%))	40 (30%)	84 (31%)	7 (18%)	1 (14%)	0 (0%)	29 (18%)	161 (26%)	
Undergraduate (n (%))	39 (29%)	71 (26%)	9 (24%)	1 (14%)	0 (0%)	57 (35%)	177 (29%)	
High school (n (%))	31 (23%)	65 (24%)	11 (29%)	1 (14%)	0 (0%)	19 (12%)	127 (21%)	
Did not complete high school n (%)	6 (< 1%)	5 (< 1%)	2 (5%)	0 (0%)	0 (0%)	1 (< 1%)	14 (< 1%)	
Ethnicity								0.037
Non-White (n (%))	9 (7%)	46 (17%)	4 (11%)	2 (29%)	0	19 (12%)	80 (13%)	
White (n (%))	126 (93%)	227 (83%)	34 (89%)	5 (71%)	1 (100%)	142 (88%)	535 (87%)	
KINDL questionnaire								
Kid KINDL (n (%))	118 (87%)	191 (70%)	21 (55%)	6 (86%)	1 (100%)	123 (76%)	460 (75%)	< 0.001
Kiddo KINDL (n (%))	17 (13%)	82 (30%)	17 (45%)	1 (14%)	0 (0%)	38 (24%)	155 (25%)	< 0.001
KINDL score^a^	64.04 ± 13.16	63.75 ± 12.26	64.14 ± 12.82	68.01 ± 9.04	87.50	72.16 ± 11.15	66.13 ± 12.71	< 0.001
SWAN inattention^b^	5.49 ± 2.86	4.42 ± 2.93	2.15 ± 2.80	3.00 ± 2.58	0.00	0.16 ± 0.47	4.08 ± 3.27	< 0.001
SWAN Hyperactivity^c^	4.13 ± 3.13	3.36 ± 3.04	0.88 ± 1.61	2.43 ± 1.62	0.00	0.08 ± 0.46	3.03 ± 3.09	< 0.001
SWAN Inattentive or Hyperactivity ≥ 6^b,c^ (n(%))	83 (62%)	127 (47%)	5 (15%)	1 (14%)	0 (0%)	0 (0%)		< 0.001
TOCS^d^	− 26.74 ± 27.55	− 11.92 ± 24.11	18.62 ± 18.72	− 25.86 ± 27.37	12.00	− 40.85 ± 23.77	− 19.13 ± 28.52	< 0.001
SCQ^e^	7.67 ± 6.33	19.27 ± 7.33	5.37 ± 4.11	4.29 ± 5.47	4.00	2.32 ± 2.25	11.23 ± 9.50	< 0.001
SCQ ≥ 11^e^	35 (26%)	234 (87%)	5 (13%)	1 (14%)	0 (0%)	2 (1%)	227 (37%)	< 0.001
IQ^f^	97.50 ± 14.65	92.28 ± 24.13	113.16 ± 11.87	103.29 ± 8.64	118.00	109.71 ± 12.13	99.45 ± 20.39	< 0.001
ABAS GAC^g^	78.90 ± 16.95	65.47 ± 15.77	93.13 ± 17.30	87.57 ± 16.72	120.00	103.98 ± 12.80	80.55 ± 22.25	< 0.001
CBCL internalizing T-Score^h^	61.79 ± 11.01	63.79 ± 9.90	64.34 ± 8.95	51.29 ± 8.52	67.00	46.95 ± 9.10	58.84 ± 12.23	< 0.001
CBCL Externalizing T-Score^i^	59.96 ± 12.04	57.96 ± 10.55	52.71 ± 10.57	52.14 ± 11.57	52.00	43.10 ± 8.47	54.11 ± 12.40	< 0.001

^a^KIDNL Range (0–100).

^b^SWAN Inattention Range (− 18 to 18) ≥ 6 Clinical Cut-off.

^c^SWAN Hyperactivity Range (− 18 to 18), ≥ 6 Clinical Cut-off.

^d^TOCS Range (− 23 to 23).

^e^SCQ Range (0–39), ≥ 11 Clinical Cut-off.

^f^IQ Range (0–144 +).

^g^ABAS GAC Range (40–120).

^h^CBCL Internalizing T-Score Range (0–100) ≥ 64 Clinical Cut-off.

^i^CBCL Externalizing T-Score (0–100) ≥ 64 Clinical Cut-off.

^j^(1) continuous normal distributed, p-value derived from ANOVA, (2) continuous non-normal, p-value derived from Kurskall-Wallis test, (3) categorical, p-value derived from Fisher Exact test.

### Pairwise correlations

**Figure 3:**
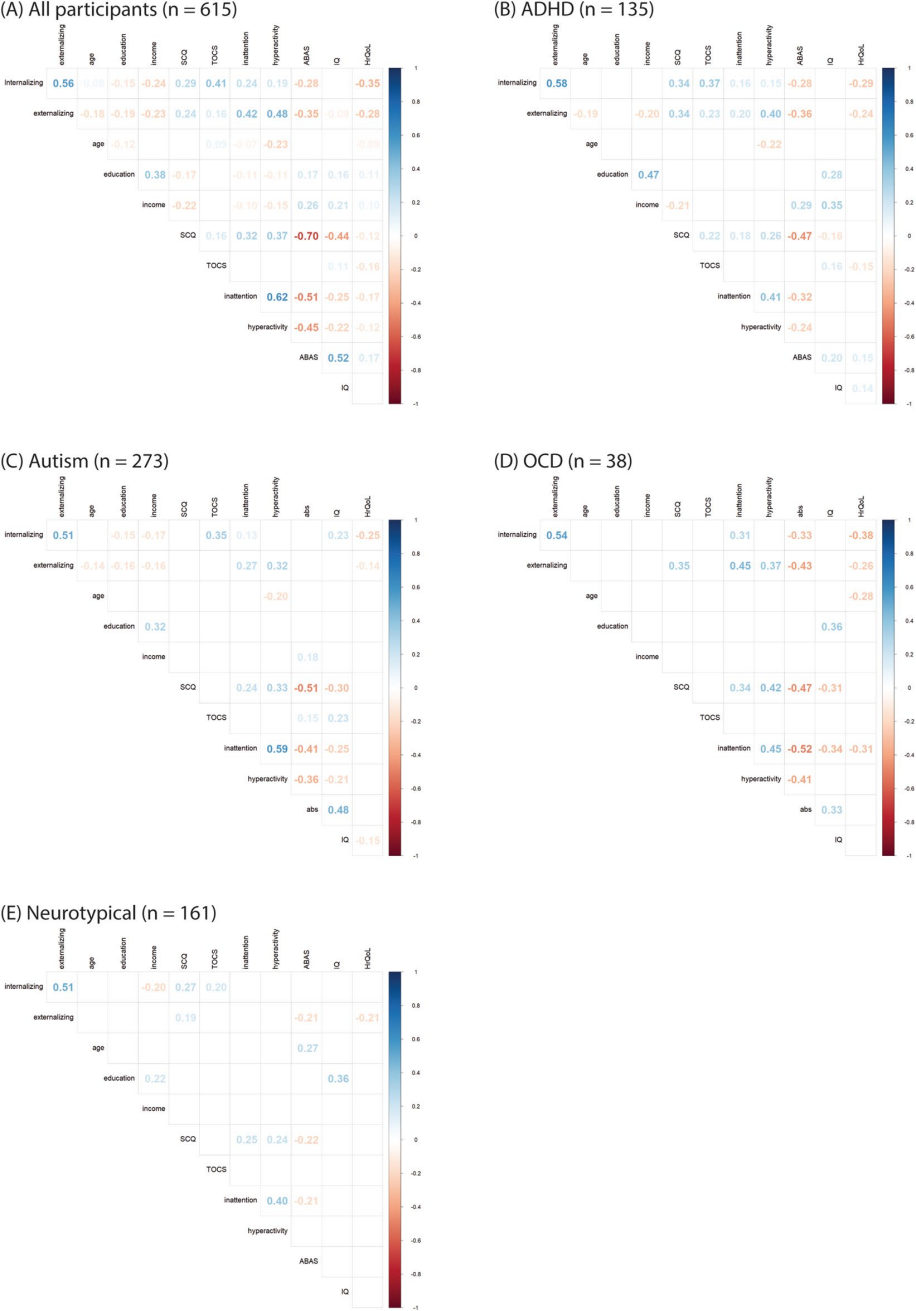
Correlation Matrix for (**A**) Total (**B**) ADHD, (**C**) autism, (**D**) OCD (**E**) TD.

**Figure 4:**
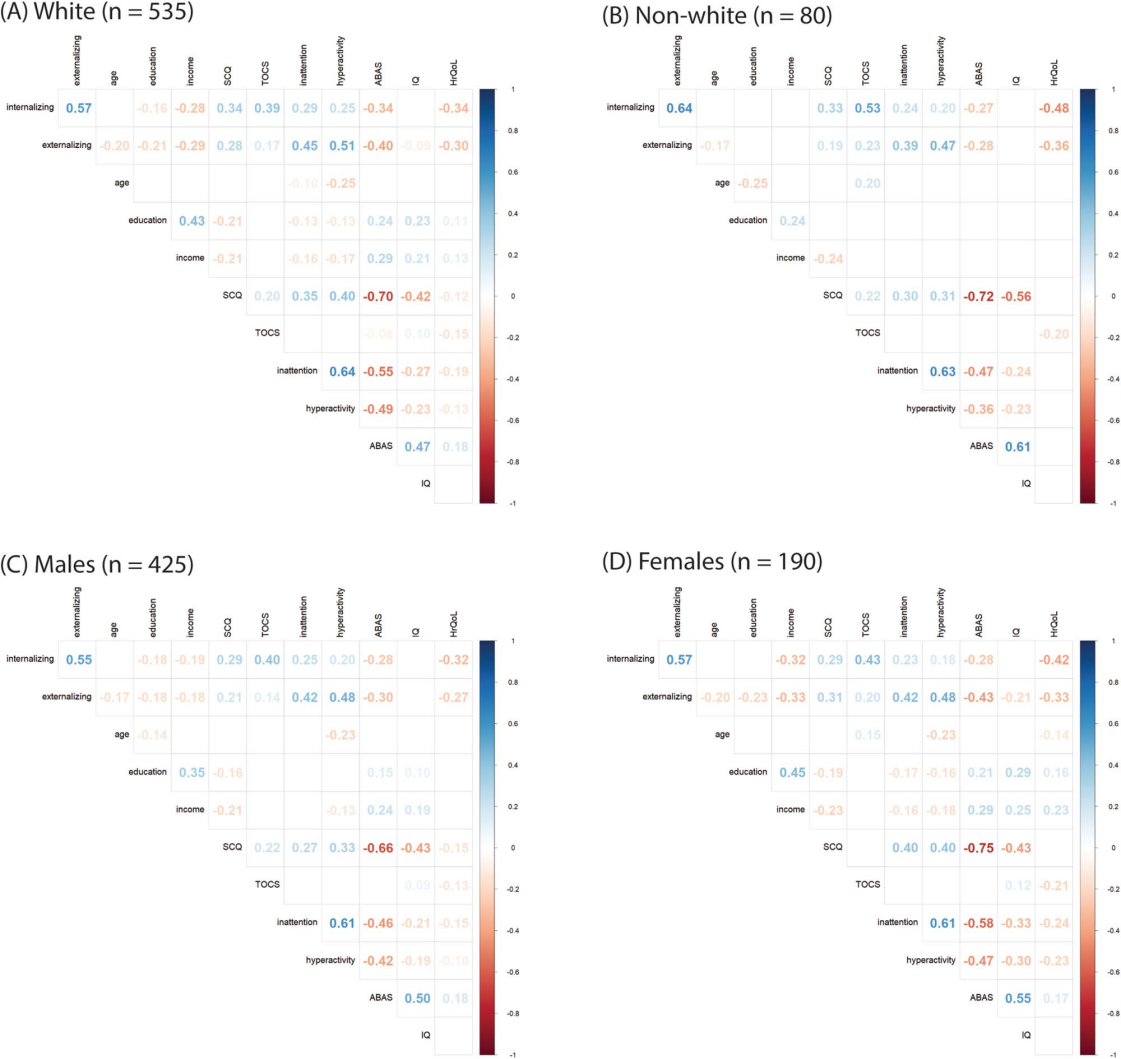
Correlation matrices by race/ethnicity: (**A**) white (**B**) non-white and sex: (**C**) male (**D**) female.

Figures 3 and 4 depicts pairwise Pearson correlations among the model variables. Across the entire participant pool, medium correlations were found between HrQoL and internalizing (r = − 0.35, p < 0.001) and externalizing symptoms (r = − 0.28, p < 0.001), while small correlations were found with adaptive functioning (r = 0.17, p < 0.001), autism traits (r = − 0.12, p < 0.001), hyperactivity (r = − 0.12, p < 0.001), inattention (r = − 0.17, p < 0.001), obsessive–compulsive symptoms (r = − 0.16, p < 0.001), parental education (r = 0.11, p < 0.001), income (r = 0.10, p < 0.001), and age (r = − 0.09, p = 0.001).

### Structural equation modelling

When the entire participant pool was used, the structural equation model fit was good to excellent (SRMR = 0.06, normed Chi-square = 4.428 TLI = 0.932, CFI = 0.956, and RMSEA = 0.075). The model explained 17.9% of variance in HrQoL in our sample.

**Figure 5:**
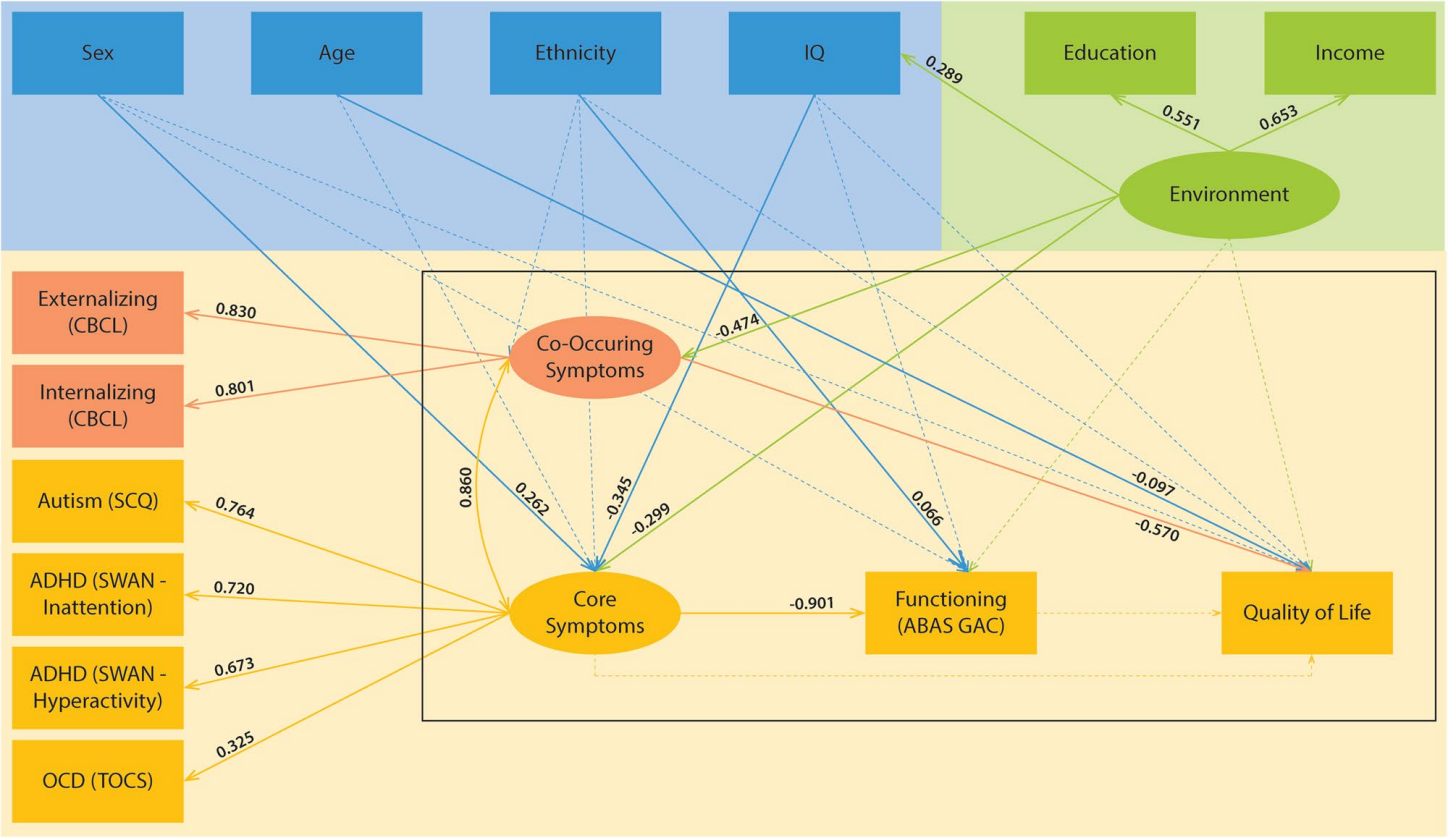
SEM Model for HrQoL. Solid lines represent significant pathways, dotted lines represent insignificant pathways.

The results are shown in Fig. 5. In this model, HrQoL was negatively associated with co-occurring symptoms (B = − 0.6, SE = 0.20, CI (− 0.95, − 0.19), p = 0.004) and age (B = − 0.1, SE = 0.04, CI (− 0.19, − 0.01), p = 0.037). The direct effects of functioning, environment, sex, ethnicity, or IQ on HrQoL were not statistically significant. Co-occurring symptoms were negatively associated with SES (B = − 0.5, SE = − 0.05, CI (− 0.58, − 0.37), p < 0.001), and correlated with core symptoms (B = 0.9, SE = 0.04, CI (0.79, 0.94), p < 0.001). This correlation with core symptoms was expected, given the overlap between externalizing behaviours and ADHD symptoms, and internalizing behaviours and OCD symptoms (e.g., anxiety).

Greater adaptive functioning was significantly associated with decreased core symptoms (B = − 0.9, SE = 0.155, CI (− 1.20, − 0.60), p < 0.001) and white ethnicity (B = 0.1, SE = 0.03, CI (0.002, 0.13), p = 0.043). Decreased core symptoms were associated with female sex (B = 0.3, SE = 0.04, CI (0.18, 0.34), p < 0.001), higher IQ (B = − 0.3, SE = 0.04, CI (− 0.42, − 0.27), p < 0.001), and greater SES (B = − 0.3, SE = 0.05, CI (− 0.40, − 0.18.9) p < 0.001). IQ was positively associated with SES (B = 0.3, SE = 0.05, CI (0.19, 0.38) p < 0.001).

To further investigate the effect of sex, the model was run separately for male/female. For the sex analysis, the model accounted for 57% and 14% of variance in HrQoL for the female and male groups, respectively, but the overall conclusions remained unchanged (Additional File 4).

## Discussion

The purpose of this study was to characterize the correlates of HrQoL in a pediatric neurodiverse population. We constructed a model based on the Revised Wilson and Cleary framework. To our knowledge, this study is the first HrQoL study using a transdiagnostic approach across neurodevelopmental conditions.

Our model explained less than 20% of the variance in HrQoL for the overall sample. This variation is in line with other studies that measured HrQoL in clinical pediatric populations^74^. Given that most of the variability in HrQoL is not accounted for by the variables included in our model, future studies are needed to understand what other factors may contribute to QoL. These factors may include the general health perceptions and biology/physiology of the Wilson and Cleary’s model of HrQoL as well as a measure to quantify the gap between one’s experience and expectation of life^75^.

Contrary to our hypothesis, only co-occurring conditions and age were directly associated with HrQoL. The association between HrQoL and externalizing/internalizing symptoms remained consistent when stratifying the sample by sex. Internalizing and externalizing behaviours have been previously associated with QoL^21^ and one’s subjective well-being^76^. These results are consistent with the findings of a recent systematic review which highlighted the negative impact of mental health symptoms on HrQoL in neurodevelopmental conditions^15^, as well as the results of a study examining perspectives of autistic adults on quality of life^77^. Mental health symptoms, including externalizing and internalizing behaviours, are also transdiagnostic domains which are impacted across neurodivergent groups^78–81^. In this context, our results highlight the strength of a transdiagnostic approach to understanding the predictors of QoL. Future studies comparing neurodiverse groups with matched participants of internalizing/externalizing behaviours and no neurodevelopmental condition can further clarify the interactions among neurodevelopmental differences, mental health conditions, and HrQoL.

Unlike previous studies^9,82,83^, we did not find a direct association between HrQoL, functioning, and core symptoms, but our results suggest that these domains may indirectly impact QoL through affecting internalizing and externalizing behaviours. This is consistent with previous findings that anxiety is a moderator of the association between autism features and quality of life^84^. It is also important to note that these findings should be interpreted in the context that our measures of symptoms and adaptive functioning reflect the perception of caregiver informants whereas our QoL measure reflects the participants’ subjective experiences.

Our model also examined the association of sociodemographic factors with HrQoL. The results suggest a direct and negative association between age and HrQoL. This is consistent with the existing literature on the association of age and QoL in neurodivergent children^9,14,16^. This association may reflect the age-related differences in societal expectations, stressors, perceptions of disability, and support^85^. These findings should be interpreted with caution given the cross sectional nature of our data. It is also interesting to note that when stratifying the participant pool by diagnosis and sex, the pairwise correlation between HrQoL and age was only significant for female participants and those with a diagnosis of OCD. Future studies are needed to understand the differential contribution of age across demographic and diagnostic subgroups.

Our structural equation model did not reveal a significant direct association between HrQoL and sex; however, our model suggests that sex may influence HrQoL indirectly through modulation of symptoms. This finding should be interpreted in the context that neurodevelopmental condition-related symptoms may present differently in girls and that our measures may be limited in capturing these differences.

We also found that higher socioeconomic status was indirectly associated with increased HrQoL, through internalizing and externalizing behaviour. The connection between these co-occurring symptoms and SES is well established in previous literature^53^ as lower SES may reflect the greater likelihood of negative life events; for example, parental employment stress or housing challenges^86^. Additionally, children from higher SES households may have greater access to mental health services. Since children with disabilities typically have increased healthcare and educational costs^87^, the socioeconomic barrier to accessing mental health services is exacerbated.

Our results did not show a significant direct or indirect association between HrQoL and race/ethnicity, although this variable is a well-established correlate of health and HrQoL^30^. Our findings should be interpreted with caution given the large imbalance in sample sizes for the white and racialized groups and the dichotomization of the race/ethnicity variable into white and non-white categories as this approach does not capture the diversity of experience in the racialized group. Given the many systemic barriers experienced by racialized families, it is critical future studies include diverse participant samples so that the diversity of experiences can be characterized.

### Limitations

A limitation of our study is the cross-sectional nature. One’s experiences, needs, supports, and environment and thus HrQoL may change significantly over the lifespan and a longitudinal analysis of predictors is needed to capture these changes. In particular, future longitudinal studies on predictors of HrQoL are highly needed.

Another limitation of this study is the construct of HrQoL used as the outcome as this measure is based on neurotypical notions of life quality. An example of this is the social wellbeing subscale which includes having and spending time with friends, a construct that may not be predictive of HrQoL for some autistic individuals. Instead, other people’s knowledge and acceptance of autism, support and services, and sensory differences may be more relevant to the QoL construct in autism^77^. The development of a subjective HrQoL measure that can be applied across neurodiverse populations is critically needed.

Thirdly, the ability to capture distinguishable co-occurring symptoms in the neurodiverse groups was limited by the instruments used. As some externalizing and internalizing behaviours can overlap with ADHD and OCD respectively, this may have hampered the ability of our model to capture distinct relationships between HrQoL and co-occurring symptoms. Further, future studies comparing predictors of HrQoL in neurodivergent populations with those in individuals with psychiatric conditions can further clarify the role of mental health symptoms.

Additionally, our analysis focused on sex and did not include gender and our participant pool did not include gender-diverse participants. Our coding of ethnicity may not reflect the heterogeneity of ethnicities white versus non-white groups. Finally, other than our diagnostic tools, the variables used in the study were based on parent-report questionnaires. Future studies with clinician-administered measures are likely to add greater understanding.

The study has several strengths. First, this study used a self-report measure of HrQoL. This is important as parental reports may be subject to biases due to the negative perceptions of symptoms^28^. Second, we used a transdiagnostic and dimensional approach to identifying correlates for HrQoL in children with autism, ADHD, OCD, and typical development. Through combining the data from participants across these groups and using core symptom domains rather than the primary diagnosis in the SEM model, we investigated the impact of different symptom domains on HrQoL, rather than the effect of diagnostic criteria. Third, the methodology of this study provided a unique and comprehensive way to understand predictors of HrQoL. The use of an SEM model enabled us to base our analysis on a theoretical model for HrQoL and create latent constructs for unobservable variables. Altogether, this created a thorough model accounting for the interrelationships between numerous variables. The variables used in our study were not restricted to functioning or symptoms, but also included a range of demographic and environmental variables to create a profile of participants in our study outside of their diagnosis.

## Conclusion

Our results suggest internalizing and externalizing symptoms are key contributors to HrQoL in children with neurodevelopmental conditions, highlighting the need for consideration of transdiagnostic symptom domains in assessment and provision of interventions and supports.

### Supplementary Information

Supplementary Information 1.

Supplementary Information 2.

Supplementary Information 3.

Supplementary Information 4.

Supplementary Information 5.

## Data Availability

Participants were drawn from the Province of Ontario Neurodevelopmental Disorders (POND) network (exported July 2021). Data from the POND network is available via a controlled data release through Ontario Brain Institute’s Brain-CODE: https://www.braincode.ca/).

## Abbreviations

ABAS: Adaptive Behaviour Assessment System
ADHD: Attention-deficit/hyperactivity disorder
ADOS: Autism Diagnostic Observation Schedule
ASD: Autism spectrum disorder
CBCL: Child Behaviour Checklist
CFI: Comparative fit index
GAC: General Adaptive Composite
HRQoL: Health-related quality of life
OCD: Obsessive–compulsive disorder
PICS: Parent Interview for Child Symptoms
POND: Province of Ontario Neurodevelopmental Disorders Network
QoL: Quality of life
SEM: Structural equation modelling
SES: Socioeconomic status
RMSEA: Root mean square error of approximation
SCQ: Social Communication Questionnaire
SRMR: Standardized root mean squared residual
SWAN: Strengths and Weaknesses of ADHD-Symptoms and Normal Behavior
TD: Typical development
TLI: Tucker Lewis index
TOCS: Toronto Obsessive Compulsive Scale
WLSMV: Weighted least square estimator

## References

[1] Bottema-Beutel K, Kapp SK, Lester JN, Sasson NJ, Hand BN (2021). Avoiding Ableist Language: Suggestions for autism researchers. Autism Adulthood.

[2] American Psychiatric Association (2013). Neurodevelopmental Disorders. Diagnostic and Statistical Manual of Mental Disorders.

[3] Kushki A (2019). Examining overlap and homogeneity in ASD, ADHD, and OCD: A data-driven, diagnosis-agnostic approach. Transl. Psychiatry.

[4] Lai M-C (2019). Prevalence of co-occurring mental health diagnoses in the autism population: A systematic review and meta-analysis. Lancet Psychiatry.

[5] Kushki A (2021). Cross-diagnosis structural correlates of autistic-like social communication differences. Cereb. Cortex.

[6] Choi EJ (2020). Beyond diagnosis: Cross-diagnostic features in canonical resting-state networks in children with neurodevelopmental disorders. NeuroImage Clin..

[7] Vandewouw MM (2023). Identifying replicable subgroups in neurodevelopmental conditions using resting-state functional magnetic resonance imaging data. JAMA Netw. Open.

[8] Becker A, Roessner V, Breuer D, Döpfner M, Rothenberger A (2011). Relationship between quality of life and psychopathological profile: Data from an observational study in children with ADHD. Eur. Child Adolesc. Psychiatry.

[9] Kuhlthau K (2010). Health-related quality of life in children with autism spectrum disorders: Results from the autism treatment network. J. Autism Dev. Disord..

[10] Lack CW (2009). Quality of life in children and adolescents with obsessive-compulsive disorder: Base rates, parent–child agreement, and clinical correlates. Soc. Psychiatry Psychiatr. Epidemiol..

[11] World Health Organization (2004). The world health organization quality of life (WHOQOL)-BREF.

[12] Bullinger M, Anderson R, Cella D, Aaronson N (1993). Developing and evaluating cross-cultural instruments from minimum requirements to optimal models. Qual. Life Res..

[13] Solomon M, Miller M, Taylor SL, Hinshaw SP, Carter CS (2012). Autism symptoms and internalizing psychopathology in girls and boys with autism spectrum disorders. J. Autism Dev. Disord..

[14] Oakley BF (2021). How do core autism traits and associated symptoms relate to quality of life? Findings from the Longitudinal European Autism Project. Autism Int. J. Res. Pract..

[15] Mahjoob M (2023). Predictors of health-related quality of life in neurodivergent children: A systematic review. Clin. Child Fam. Psychol. Rev..

[16] Storch EA (2018). Quality of life in children and youth with obsessive-compulsive disorder. J. Child Adolesc. Psychopharmacol..

[17] Guzick AG (2019). Parents’ perceptions of internalizing and externalizing features in childhood OCD. Child Psychiatry Hum. Dev..

[18] Kuja-Halkola R, Lichtenstein P, D’Onofrio BM, Larsson H (2015). Codevelopment of ADHD and externalizing behavior from childhood to adulthood. J. Child Psychol. Psychiatry.

[19] Luijten CC, van de Bongardt D, Jongerling J, Nieboer AP (2021). Associations between adolescents’ internalizing problems and well-being: is there a buffering role of boys’ and girls’ relationships with their mothers and fathers?. BMC Public Health.

[20] Weeks M (2016). Developmental pathways linking childhood and adolescent internalizing, externalizing, academic competence, and adolescent depression. J. Adolesc..

[21] Williamson AA (2021). Sleep problems, internalizing and externalizing symptoms, and domains of health-related quality of life: Bidirectional associations from early childhood to early adolescence. Sleep.

[22] Evans S, Sciberras E, Mulraney M (2020). The relationship between maternal stress and boys’ ADHD symptoms and quality of life: An Australian prospective cohort study. J. Pediatr. Nurs. Nurs. Care Child. Fam..

[23] Payakachat N (2014). Predicting health utilities for children with autism spectrum disorders. Autism Res..

[24] Vlassoff C (2007). Gender differences in determinants and consequences of health and illness. J. Health Popul. Nutr..

[25] Smith SS (2013). The influence of stress at puberty on mood and learning: role of the α4βδ GABAA receptor. Neuroscience.

[26] Coluccia A, Ferretti F, Fagiolini A, Pozza A (2017). Quality of life in children and adolescents with obsessive-compulsive disorder: A systematic review and meta-analysis. Neuropsychiatr. Dis. Treat..

[27] Lee Y (2016). Meta-analysis of quality of life in children and adolescents with ADHD: By both parent proxy-report and child self-report using PedsQL™. Res. Dev. Disabil..

[28] van Heijst BF, Geurts HM (2015). Quality of life in autism across the lifespan: A meta-analysis. Autism.

[29] Ravens-Sieberer U, Erhart M, Wille N, Bullinger M, the BELLA study group (2008). Health-related quality of life in children and adolescents in Germany: Results of the BELLA study. Eur. Child Adolesc. Psychiatry.

[30] Wallander JL (2019). Racial/ethnic disparities in health-related quality of life and health status across pre-, early-, and mid-adolescence: A prospective cohort study. Qual. Life Res..

[31] Kuhlthau KA, McDonnell E, Coury DL, Payakachat N, Macklin E (2018). Associations of quality of life with health-related characteristics among children with autism. Autism.

[32] Baribeau DA (2015). Examining and comparing social perception abilities across childhood-onset neurodevelopmental disorders. J. Am. Acad. Child Adolesc. Psychiatry.

[33] Cullen B (2008). Social and communication difficulties and obsessive-compulsive disorder. Psychopathology.

[34] Matson JL, Rieske RD, Williams LW (2013). The relationship between autism spectrum disorders and attention-deficit/hyperactivity disorder: An overview. Res. Dev. Disabil..

[35] Zandt F, Prior M, Kyrios M (2007). Repetitive behaviour in children with high functioning autism and obsessive compulsive disorder. J. Autism Dev. Disord..

[36] Insel T (2010). Research domain criteria (RDoC): Toward a new classification framework for research on mental disorders. Am. J. Psychiatry.

[37] Lord, C. *et al. ADOS-2. Manual (Part I): Modules, 1–4*. (Western Psychological Services Torrance, 2012).

[38] Lord C, Rutter M, Le Couteur A (1994). Autism Diagnostic Interview-Revised: A revised version of a diagnostic interview for caregivers of individuals with possible pervasive developmental disorders. J. Autism Dev. Disord..

[39] Ickowicz A (2006). The parent interview for child symptoms: A situation-specific clinical research interview for attention-deficit hyperactivity and related disorders. Can. J. Psychiatry.

[40] Scahill L (1997). Children’s Yale-Brown obsessive compulsive scale: reliability and validity. J. Am. Acad. Child Adolesc. Psychiatry.

[41] Ferrans CE, Zerwic JJ, Wilbur JE, Larson JL (2005). Conceptual Model of Health-Related Quality of Life. J. Nurs. Scholarsh..

[42] Bakas T (2012). Systematic review of health-related quality of life models. Health Qual. Life Outcomes.

[43] Ravens-Sieberer, U. & Bullinger, M*. KINDLR Questionnaire for Measuring Health-Related Quality of Life in Children and Adolescents Revised Version Manual* (2000).

[44] Rutter M, Bailey A (2003). The Social Communication Questionnaire (SCQ).

[45] Swanson JM (2012). Categorical and dimensional definitions and evaluations of symptoms of ADHD: History of the SNAP and the SWAN rating scales. Int. J. Educ. Psychol. Assess..

[46] Park LS (2016). The Toronto obsessive-compulsive scale: Psychometrics of a dimensional measure of obsessive-compulsive traits. J. Am. Acad. Child Adolesc. Psychiatry.

[47] Achenbach, T. M. & Edelbrock, C. S. *Manual for the Child: Behavior Checklist and Revised Child Behavior Profile* (1983).

[48] Harrison PL, Oakland T (2000). Adaptive Behavior Assessment System.

[49] Wechsler D (1999). Wechsler Abbreviated Scale of Intelligence.

[50] Wechsler D, Kodama H (1949). Wechsler Intelligence Scale for Children.

[51] Binet A, Simon T (1961). The Development of Intelligence in Children.

[52] Wechsler D (2012). Wechsler Preschool and Primary Scale of Intelligence—Fourth Edition.

[53] Yu Y, Williams DR, Aneshensel CS, Phelan JC (1999). Socioeconomic Status and Mental Health. Handbook of the Sociology of Mental Health.

[54] Bradley RH, Corwyn RF (2002). Socioeconomic status and child development. Annu. Rev. Psychol..

[55] Holm S (1979). A simple sequentially rejective multiple test procedure. Scand. J. Stat..

[56] Broc G, Gana K (2018). Structural Equation Modeling with lavaan.

[57] Beran TN, Violato C (2010). Structural equation modeling in medical research: A primer. BMC Res. Notes.

[58] Dellapiazza F, Audras-Torrent L, Michelon C, Baghdadli A (2021). Clinical characteristics of children with ASD and comorbid ADHD: Association with social impairment and externalizing and internalizing behaviours. Res. Dev. Disabil..

[59] Istvan EM, Nevill RE, Mazurek MO (2020). Sensory over-responsivity, repetitive behavior, and emotional functioning in boys with and without autism spectrum disorder. Res. Autism Spectr. Disord..

[60] Bal VH, Kim S-H, Fok M, Lord C (2019). Autism spectrum disorder symptoms from ages 2 to 19 years: Implications for diagnosing adolescents and young adults. Autism Res..

[61] Bölte S (2023). Sex and gender in neurodevelopmental conditions. Nat. Rev. Neurol..

[62] Lahey BB, Pelham WE, Loney J, Lee SS, Willcutt E (2005). Instability of the DSM-IV subtypes of ADHD from preschool through elementary school. Arch. Gen. Psychiatry.

[63] Mayes S, Castagna P, DiGiovanni C, Waschbusch D (2020). Relationship between ADHD, oppositional defiant, conduct, and disruptive Mood dysregulation disorder symptoms and age in children with ADHD and autism. Int. J. Clin. Psychiatry Ment. Health.

[64] Chandler S (2022). Pathways to adaptive functioning in autism from early childhood to adolescence. Autism Res..

[65] Mahendiran T (2019). Sex differences in social adaptive function in autism spectrum disorder and attention-deficit hyperactivity disorder. Front. Psychiatry.

[66] Tillmann J (2019). Investigating the factors underlying adaptive functioning in autism in the EU-AIMS Longitudinal European Autism Project. Autism Res..

[67] Rosseel Y (2012). lavaan: An R package for structural equation modeling. J. Stat. Softw..

[68] Schumacker RE, Lomax RG (2004). A Beginner’s Guide to Structural Equation Modeling.

[69] Brown, T. A. *Confirmatory Factor Analysis for Applied Research* 475 (2006).

[70] Hu L, Bentler PM (1999). Cutoff criteria for fit indexes in covariance structure analysis: Conventional criteria versus new alternatives. Struct. Equ. Model. Multidiscip. J..

[71] Browne MW, Cudeck R (1992). Alternative ways of assessing model fit. Sociol. Methods Res..

[72] Li C-H (2016). Confirmatory factor analysis with ordinal data: Comparing robust maximum likelihood and diagonally weighted least squares. Behav. Res. Methods.

[73] Wolf EJ, Harrington KM, Clark SL, Miller MW (2013). Sample size requirements for structural equation models: An evaluation of power, bias, and solution propriety. Educ. Psychol. Meas..

[74] Villalonga-Olives E (2014). Pediatric health-related quality of life: A structural equation modeling approach. PLoS ONE.

[75] Calman KC (1984). Quality of life in cancer patients: An hypothesis. J. Med. Ethics.

[76] Bartels M, Cacioppo JT, van Beijsterveldt TCEM, Boomsma DI (2013). Exploring the association between well-being and psychopathology in adolescents. Behav. Genet..

[77] McConachie H (2020). What is important in measuring quality of life? Reflections by autistic adults in four countries. Autism Adulthood.

[78] Bauminger N, Solomon M, Rogers SJ (2010). Externalizing and internalizing behaviors in ASD. Autism Res..

[79] Ghanizadeh A, Mohammadi MR, Moini R (2008). Comorbidity of psychiatric disorders and parental psychiatric disorders in a sample of Iranian children with ADHD. J. Atten. Disord..

[80] Jensen P, Shervette R, Xenakis S, Richters J (1993). Anxiety and depressive disorders in attention deficit disorder with hyperactivity: New findings. Am. J. Psychiatry.

[81] Storch EA (2006). Peer victimization in children with obsessive-compulsive disorder: Relations with symptoms of psychopathology. J. Clin. Child Adolesc. Psychol..

[82] Kamp-Becker I, Schröder J, Remschmidt H, Bachmann CJ (2010). Health-related quality of life in adolescents and young adults with high functioning autism-spectrum disorder. Psycho-Soc. Med..

[83] Knüppel A, Telléus GK, Jakobsen H, Lauritsen MB (2018). Quality of life in adolescents and adults with autism spectrum disorder: Results from a nationwide Danish survey using self-reports and parental proxy-reports. Res. Dev. Disabil..

[84] Smith IC, Ollendick TH, White SW (2019). Anxiety moderates the influence of ASD severity on quality of life in adults with ASD. Res. Autism Spectr. Disord..

[85] Chiang H-M, Wineman I (2014). Factors associated with quality of life in individuals with autism spectrum disorders: A review of literature. Res. Autism Spectr. Disord..

[86] Baum A, Garofalo JP, Yali AM (1999). Socioeconomic status and chronic stress: Does stress account for SES effects on health?. Ann. N. Y. Acad. Sci..

[87] Anderson D, Dumont S, Jacobs P, Azzaria L (2007). The personal costs of caring for a child with a disability: A review of the literature. Public Health Rep. Wash. DC.

