# Supplementary material for: Predictors of health-related quality of life for children with neurodevelopmental conditions
Source: Sci Rep. 2024 Mar 16;14:6377. doi: 10.1038/s41598-024-56821-9 (PMC10944519; doi:10.1038/s41598-024-56821-9)
Supplement: Supplementary file 2 — Supplementary Information 2. [file 41598_2024_56821_MOESM2_ESM.docx]

**Additional File 2: Participant Demographics of Total Participants (Including Participants with Incomplete Data).**

ADHD: Attention-deficit Hyperactivity Disorder, ASD: Autism spectrum disorder, OCD: Obsessive Compulsive Disorder. Reported as mean ± standard deviation unless otherwise reported.

|  | ADHD | ASD | OCD | SubADHD | SubOCD | TD | Total |
| --- | --- | --- | --- | --- | --- | --- | --- |
|  | *N=541* | *N=445* | *N=154* | *N=119* | *N=6* | *N=207* | *N=1472* |
| Gender |  |  |  |  |  |  |  |
| Female (n(%)) | 141 (26%) | 99 (22%) | 71 (46%) | 52 (44%) | 4 (67%) | 87 (42%) | 454 (31%) |
| Male (n(%)) | 400 (74%) | 346 (78%) | 83 (54%) | 67 (56%) | 2 (33%) | 120 (58%) | 1018 (69%) |
| Age in Years (mean ± standard deviation) | 10.46  ± 2.57 | 11.68  ± 2.89 | 12.56  ± 2.57 | 10.22  ± 2.69 | 12.17  ± 2.86 | 11.20  ± 2.79 | 11.14  ± 2.80 |
| Income^:^ |  |  |  |  |  |  |  |
| High ($150,000) (n(%)) | 43  (23%) | 69  (21%) | 22  (32%) | 8 (57%) | 1 (100%) | 69 (39%) | 212 (27%) |
| Middle ($50,000-$150,000) | 85 (46%) | 169 (51%) | 33 (48%) | 4 (29%) | 0 (0%) | 85 (48%) | 376 (48%) |
| Low ($0-$49,999) | 56 (30%) | 96 (29%) | 14 (20%) | 2 (14%) | 0 (0%) | 24 (13%) | 192 (25%) |
| Education |  |  |  |  |  |  |  |
| Graduate/  Professional (n(%)) | 29 (15%) | 67 (17%) | 19 (23%) | 10 (59%) | 1 (100%) | 65 (32%) | 191 (21%) |
| Associate (n(%)) | 56 (28%) | 112 (29%) | 17 (20%) | 1 (6%) | 0 (0%) | 42 (21%) | 228 (26%) |
| Undergraduate (n(%)) | 59 (30%) | 105 (27%) | 26 (31%) | 4 (24%) | 0 (0%) | 69 (34%) | 263 (30%) |
| High School (n(%)) | 45 (23%) | 92 (24%) | 19 (23%) | 2 (12%) | 0 (0%) | 22 (11%) | 180 (20%) |
| Did Not Complete High School n(%) | 8 (4%) | 15 (4%) | 3 (4%) | 0 (0%) | 0 (0%) | 3 (1%) | 29 (3%) |
| Ethnicity |  |  |  |  |  |  |  |
| Non-White (n(%)) | 88 (19%) | 83 (20%) | 16 (12%) | 25 (22%) | 2 (40%) | 33 (16%) | 247 (19%) |
| White (n(%)) | 365 (81%) | 334 (80%) | 120 (88%) | 91 (78%) | 3 (60%) | 169 (84%) | 1082 (81%) |
| KINDL Questionnaire |  |  |  |  |  |  |  |
| Kid KINDL (n(%)) | 463 (86%) | 305 (69%) | 99 (64%) | 104 (87%) | 4 (67%) | 157 (76%) | 1132 (76%) |
| Kiddo KINDL (n(%)) | 78 (14%) | 140 (31%) | 55 (36%) | 15 (13%) | 2 (33%) | 50 (24%) | 340 (23%) |
| KINDL score | 63.99 ±13.99 | 63.63 ±12.36 | 63.19 ±13.03 | 65.11 ±15.45 | 64.76 ±17.13 | 72.57 ±11.28 | 65.10 ± 13.52 |
| SWAN Inattention | 5.80 ±2.85 | 4.43 ±2.96 | 1.94 ±2.73 | 3.79 ±2.68 | 0.33 ±0.52 | 0.14± 0.44 | 4.05 ± 3.29 |
| SWAN Hyperactivity | 4.16 ±3.17 | 3.53 ±3.05 | 0.95 ±1.85 | 2.20 ±2.26 | 0.83 ±2.04 | 0.10 ±0.50 | 2.93 ± 3.10 |
| TOCS | -23.20 ±26.46 | -14.23 ±25.16 | 17.68 ±19.43 | -25.47 ±26.01 | 12.00 ±17.91 | -39.37 ±24.26 | -19.48 ± 28.72 |
| SCQ | 7.88 ±6.31 | 19.32 ±7.22 | 6.27 ±5.80 | 5.11 ±4.64 | 4.83 ±3.97 | 2.62 ±2.74 | 10.20 ± 8.70 |
| IQ | 100.95 ±14.58 | 90.69 ±24.45 | 111.24 ±13.39 | 102.33 ±13.92 | 113.00 ±11.63 | 109.26 ±12.13 | 100.27 ± 19.26 |
| ABAS GAC | 79.33 ±15.91 | 64.05 ±16.42 | 93.02 ±18.16 | 87.20 ±15.34 | 102.75 ±11.59 | 103.74 ±12.75 | 80.57 ± 21.00 |
| CBCL Internalizing  T-Score | 61.20 ±11.13 | 62.97 ±10.18 | 64.03 ±10.55 | 59.03 ±11.87 | 65.50 ±6.16 | 47.67 ±9.21 | 59.94 ± 11.76 |
| CBCL Externalizing  T-Score | 61.13 ±11.23 | 57.10 ±10.64 | 51.90 ±11.28 | 55.85 ±11.40 | 55.67 ±8.21 | 43.43 ±8.62 | 56.00 ± 12.20 |
