# Supplementary material for: Predictors of health-related quality of life for children with neurodevelopmental conditions
Source: Sci Rep. 2024 Mar 16;14:6377. doi: 10.1038/s41598-024-56821-9 (PMC10944519; doi:10.1038/s41598-024-56821-9)
Supplement: Supplementary file 3 — Supplementary Information 3. [file 41598_2024_56821_MOESM3_ESM.docx]

**Additional File 3: Participant Demographics by Questionnaire Type.**

|  | Kid-KINDL | Kiddo-KINDL |
| --- | --- | --- |
|  | *N=460* | *N=155* |
| Gender |  |  |
| Female | 139 (30%) | 51 (33%) |
| Male | 321 (69%) | 104 (67%) |
| Age in Years | 9.98 ± 1.93 | 15.14 ± 1.02 |
| Income^:^ |  |  |
| High | 131 (28%) | 37 (24%) |
| Middle | 216 (47%) | 75 (48%) |
| Low | 113 (25%) | 43 (28%) |
| Education |  |  |
| Graduate/  Professional (%) | 103 (22:78) | 33 (21:79) |
| Associate (%) | 106 (23:77) | 54 (35:65) |
| Undergraduate (%) | 146 (32:68) | 31 (20:80) |
| High School (%) | 93 (20:80) | 34 (22:78) |
| Did Not Complete High School (%) | 11 (2:98) | 5 (2:98) |
| Ethnicity |  |  |
| Non-White | 58 (12%) | 22 (14%) |
| White | 402 (87%) | 133 (85%) |
| Diagnosis |  |  |
| ADHD | 118 (87%) | 17 (11%) |
| ASD | 191 (42%) | 82 (53%) |
| OCD | 21 (5%) | 17 (11%) |
| Sub-threshold ADHD | 6 (1%) | 1 (1%) |
| Sub-threshold OCD | 1 (0%) | 0 (0%) |
| Typically Developing | 123 (27%) | 38 (25%) |

ADHD: Attention-deficit Hyperactivity Disorder, ASD: Autism spectrum disorder, OCD: Obsessive Compulsive Disorder. Reported as mean ± standard deviation unless otherwise reported.
