# Supplementary material for: Predictors of health-related quality of life for children with neurodevelopmental conditions
Source: Sci Rep. 2024 Mar 16;14:6377. doi: 10.1038/s41598-024-56821-9 (PMC10944519; doi:10.1038/s41598-024-56821-9)
Supplement: Supplementary file 4 — Supplementary Information 4. [file 41598_2024_56821_MOESM4_ESM.docx]

**Additional File 4: Sub-group Analysis of SEM Models.**

|  | Normed Chi-square | SRMR | RMSEA | CFI | TLI | R^2^ |
| --- | --- | --- | --- | --- | --- | --- |
|  |  |  |  |  |  |  |
| Sup-Group Analysis of Original SEM Model |  |  |  |  |  |  |
| Female | 1.48 | 0.05 | 0.05 | 0.99 | 0.98 | 0.57 |
| Male | 3.75 | 0.07 | 0.08 | 0.95 | 0.92 | 0.14 |
