# Supplementary material for: Predictors of health-related quality of life for children with neurodevelopmental conditions
Source: Sci Rep. 2024 Mar 16;14:6377. doi: 10.1038/s41598-024-56821-9 (PMC10944519; doi:10.1038/s41598-024-56821-9)

**Additional File 5: Flowchart of breakdown of the excluded sample based on availability of demographic, phenotypic, and socioeconomic data.**
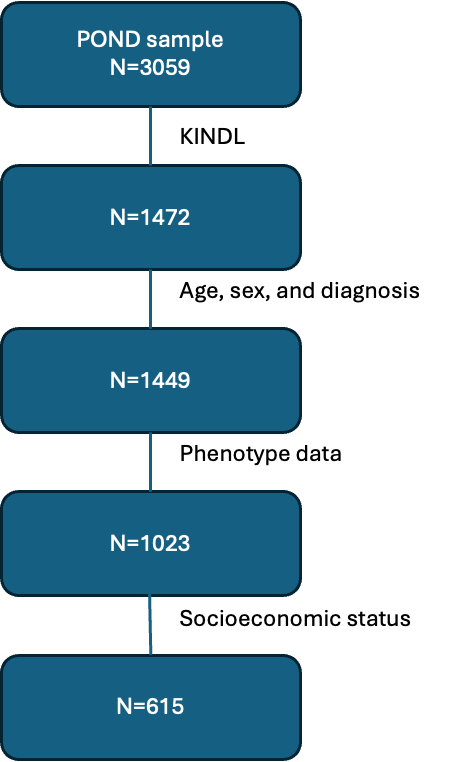

Supplement: Supplementary file 5 — Supplementary Information 5. [file 41598_2024_56821_MOESM5_ESM.docx]
